# Constitutive Activation of an Anthocyanin Regulatory Gene *PcMYB10*.*6* Is Related to Red Coloration in Purple-Foliage Plum

**DOI:** 10.1371/journal.pone.0135159

**Published:** 2015-08-06

**Authors:** Chao Gu, Liao Liao, Hui Zhou, Lu Wang, Xianbao Deng, Yuepeng Han

**Affiliations:** 1 Key Laboratory of Plant Germplasm Enhancement and Specialty Agriculture, Wuhan Botanical Garden of the Chinese Academy of Sciences, Wuhan, Hubei, People’s Republic of China; 2 Graduate University of Chinese Academy of Sciences, 19A Yuquanlu, Beijing, P.R. China; Key Laboratory of Horticultural Plant Biology (MOE), CHINA

## Abstract

Cherry plum is a popular ornamental tree worldwide and most cultivars are selected for purple foliage. Here, we report the investigation of molecular mechanism underlying red pigmentation in purple-leaf plum ‘Ziyeli’ (*Prunus cerasifera* Ehrhar f. atropurpurea (Jacq.) Rehd.), which shows red color pigmentation in fruit (flesh and skin) and foliage. Six anthocyanin-activating MYB genes, designated *PcMYB10*.*1* to *PcMYB10*.*6*, were isolated based on RNA-Seq data from leaves of cv. Ziyeli. Of these *PcMYB10* genes, five (*PcMYB10*.*1* through *PcMYB10*.*5*) show distinct spatial and temporal expression patterns, while the *PcMYB10*.*6* gene is highly expressed in all the purple-coloured organs of cv. Ziyeli. Constitutive activation of *PcMYB10*.*6* is closely related to red pigmentation in the leaf, fruit (flesh and skin), and sepal. However, the *PcMYB10*.*6* activation cannot induce red pigmentation in the petal of cv. Ziyeli during late stages of flower development due to due to a lack of expression of *PcUFGT*. The inhibition of red pigmentation in the petal of cherry plum could be attributed to the high-level expression of *PcANR* that directs anthocyanidin flux to proanthocyanidin biosynthesis. In addition, *PcMYB10*.*2* is highly expressed in fruit and sepal, but its expression cannot induce red pigmentation. This suggests the *PcMYB10* gene family in cherry plum may have diverged in function and *PcMYB10*.*2* plays little role in the regulation of red pigmentation. Our study provides for the first time an example of constitutive activation of an anthocyanin-activating *MYB* gene in *Prunus* although its underlying mechanism remains unclear.

## Introduction

Anthocyanins are an important and widespread group of pigments that confer the red, purple and blue colours to various plant organs such as flowers, leaves, and fruits. Anthocyanin pigmentation not only has an important effect on the appearance of fruits, but is also an important contributor to ornamental value of landscape plants. Beside their role in pigmentation, anthocyanins are also beneficial to human health as they act as powerful antioxidants to protect the body's cells from the potential damage caused by free radicals [1]. Fruit trees not only provide delicious fruit, but can also serve an ornamental purpose. Thus, the regulatory mechanisms of anthocyanin biosynthesis have also been extensively investigated in fruit trees, such as grape [2], apple [3–8], Chinese bayberry [9], pear [10–13], kiwifruit [14], mangosteen [15], orange [16], and peach [17, 18].

The biosynthesis of anthocyanins is catalyzed by several enzymes, including chalocone synthase (CHS), chalocone isomerase (CHI), flavanone 3-hydroxylase (F3H), flavonoid 3’-hydroxylase (F3’H), flavonoid 3’, 5’-hydroxylase (F3’5’H), dihydroflavonol 4-reductase (DFR), leucoanthocyanidin dioxygenase (LDOX), UDP-flavonoid glucosyl transferase (UFGT). Although some plants such as *Arabidopsis*, apple, and peach do not have functional F3’5’H enzymes [19–21], the anthocyanin biosynthetic pathway is highly conserved among different plant species [22, 23]. Increasing evidence suggests that the enzymes in anthocyanin biosynthetic pathway, which are anchored in the endoplasmic reticulum membrane, are likely to form a metabolon via protein-protein interaction [24, 25]. The structural genes in anthocyanin biosynthetic pathway are coordinately regulated at transcriptional level by a MBW complex comprising of three types of transcription factors (TFs), R2R3 MYB, basic helix-loop-helix (bHLH) and WD40-repeat proteins [26, 27]. Mutations in anthocyanin structural genes or transcriptional regulators can inhibit anthocyanin accumulation [28–30]. In addition, proanthocyanidin biosynthesis enzymes, leucoanthocyanidin reductase (LAR) and anthocyanidin reductase (ANR), also influence the synthesis of anthocyanin by competing with ANS and UFGT for directing leucoanthocyanidin and anthocyanidin substrates flux to proanthocyanidin biosynthetic pathway, a branch of anthocyanin biosynthesis pathway [31–34]. Therefore, the biosynthesis of anthocyanins is not yet fully understood although anthocyanin structural and regulatory genes have been identified in a variety of plant species.

Cherry plum (*Prunus ceracifera* Ehrh.), a species of plum, belongs to the genus *Prunus* that has a basic chromosome number x = 8, within the family Rosaceae [35]. The cherry plum is a popular ornamental tree worldwide, and most cultivars are selected for purple foliage although some varieties have fruits that can be eaten fresh or used for jam making. The purple-foliage plum is often called purple-leaf plum. Like the red-fleshed apple ‘Red Field’ [5], the purple-leaf plum is red-pigmented throughout all tissues including leaves and fruit (skin and flesh), and its red coloration is also due to the accumulation of anthocyanins [36, 37]. To date, there is no report on the molecular mechanism underlying red coloration in purple-leaf plum.

In the Rosaceae family, anthocyanin accumulation is controlled primarily via transcriptional regulation by MYB-type anthocyanin regulators, and their spatial and temporal expression profiles are closely related to the diverse patterns of anthocyanin pigmentation [37]. Our recent study further indicates that anthocyanin-activating MYB genes in Rosaceae can be divided into two families MYBI and MYBII [38]. The former is mainly responsible for anthocyanin accumulation in fruits, whereas the later controls anthocyanin pigmentation in vegetative organs. In this study, we report on the identification of anthocyanin-activating *MYB* genes in purple-leaf plum, and their expression profiles were also examined in different organs, including leaves, flowers, and fruits. The objective of this study is to identify anthocyanin MYB regulator(s) responsible for red pigmentation in purple-leaf plum.

## Materials and Method

### Plant materials

A purple-leaf plum ‘Ziyeli’ and a green-leaved cherry plum ‘Aoben’ were selected for this study and they are maintained at Wuhan Botanical Garden of the Chinese Academy of Sciences (Wuhan, Hubei Province, China). Flower samples were collected at the pink, bloom, and full bloom stages, while leave samples were collected every fifteen days from 5 to 110 days after full bloom (DAFB). Fruit samples were collected every five days from 30 to 60 DAFB, and each accession had three replicates, consisting of 5 fruits. The fruits were separated into flesh and peel, and the flesh was cut into small pieces. Flesh or peel from each biological replicate was mixed. All samples were immediately frozen in liquid nitrogen after collection or treatment and then store at -80°C until use.

### cDNA library preparation and Illumina sequencing

Total RNA was extracted from leaves using the Trizol reagent, followed by RNA cleanup using RNase-free DNaseI (Takara, Dalian, China). PolyA mRNAs were purified using oligo-dT-attached magnetic beads. The purified mRNAs were cleaved into small pieces (200 ~ 500 bp) by super sonication, and then subjected to first- and second-strand cDNA synthesis using random hexamer primers. RNA-Seq library was prepared according to protocols of Illumina gene expression sample preparation kit. The main reagents and supplies are Illumina Gene Expression Sample Prep Kit and Illumina Sequencing Chip (flowcell), and transcriptome sequencing was conducted using Illumina HiSeqTM 2000 System.

### Mapping RNA-Seq reads to the peach genome

Sequencing-received raw image data were transformed into sequence data. The raw data or reads were stored in fastq format, and then subjected to process through in-house perl scripts. In this step, clean data (clean reads) were obtained by removing reads containing adapter, reads containing poly-N and low quality reads from raw data. At the same time, error rate of sequence of clean data were estimated based on the Phred score (Q_phred_) and GC content. All clean reads were mapped to the peach reference genome sequences (http://www.rosaceae.org) and 3 bp mismatches could be considered at most. Index of the reference genome was built using Bowite V2.0.6 and single-end clean reads were aligned to the reference genome using TopHat v 2.0.9. Overlapping clean reads were merged into continuous transcribed sequences using cufflink package [39], and the splice junction maps and splicing isoforms were simultaneously generated.

### Isolation of genes involved in anthocyanin biosynthesis and phylogenetic analysis

Local BLASTN was performed using BioEdit software (http://www.mbio.ncsu.edu/BioEdit/bioedit.html) to identify anthocyanin structural genes and MYB-type anthocyanin regulators from transcriptome database of cherry plum. The recovered genes involved in anthocyanin biosynthesis were confirmed by comparing their nucleotide sequences against the NCBI non-redundant nucleotide (NR) database (http://blast.ncbi.nlm.nih.gov/Blast.cgi). Phylogenetic tree was conducted by Molecular Evolutionary Genetics Analysis (MEGA) software version 5 using neighbor-joining method, and sequence alignment was performed using CLUSTAL X program [40].

### Quantitative real-time RT-PCR (qRT-PCR) analysis

Total RNA was extracted using Green SPIN plant RNA extraction kit (Zomanbio, Beijing, China) according to the manufacturer’s instructions. RNA samples were treated with DNase I (TaKaRa, Dalian, China) to remove DNA contamination. The integrity of total RNA samples was assessed by electrophoresis and their concentration was examined using a NanoDrop Lite Spectrophotometer (Thermo Scientific, CH, USA). A total of 3 μg total RNA per sample was used to cDNA synthesis using Superscript III reverse transcriptase (Invitrogen). The qRT-PCR assay was carried out in a total volume of 20 μL reaction mixture containing 10 μL of 2 × SYBR Green I Master Mix (TaKaRa), 0.2 μM of each primer, and 100 ng of template cDNA. qRT-PCR was performed using a 7500 fast Real-time PCR System (Applied Biosystems). The amplification program consisted of 1 cycle of 95°C for 1 min, 40 cycles of 95°C for 10 s, and 60°C for 30 s. In addition, reaction mixtures without cDNA templates were also run as negative control, and all analyses were repeated three times using biological replicates. A cherry plum *GAPDH* gene (GenBank accession no. KP765685) was used as an endogenous control gene as its expression level was stable in different tissues throughout development (S1 Fig). All the primers are listed in S1 Table.

## Results

### Anthocyanin accumulation in two cherry plum cv. Ziyeli and Aoben

**Fig 1:**
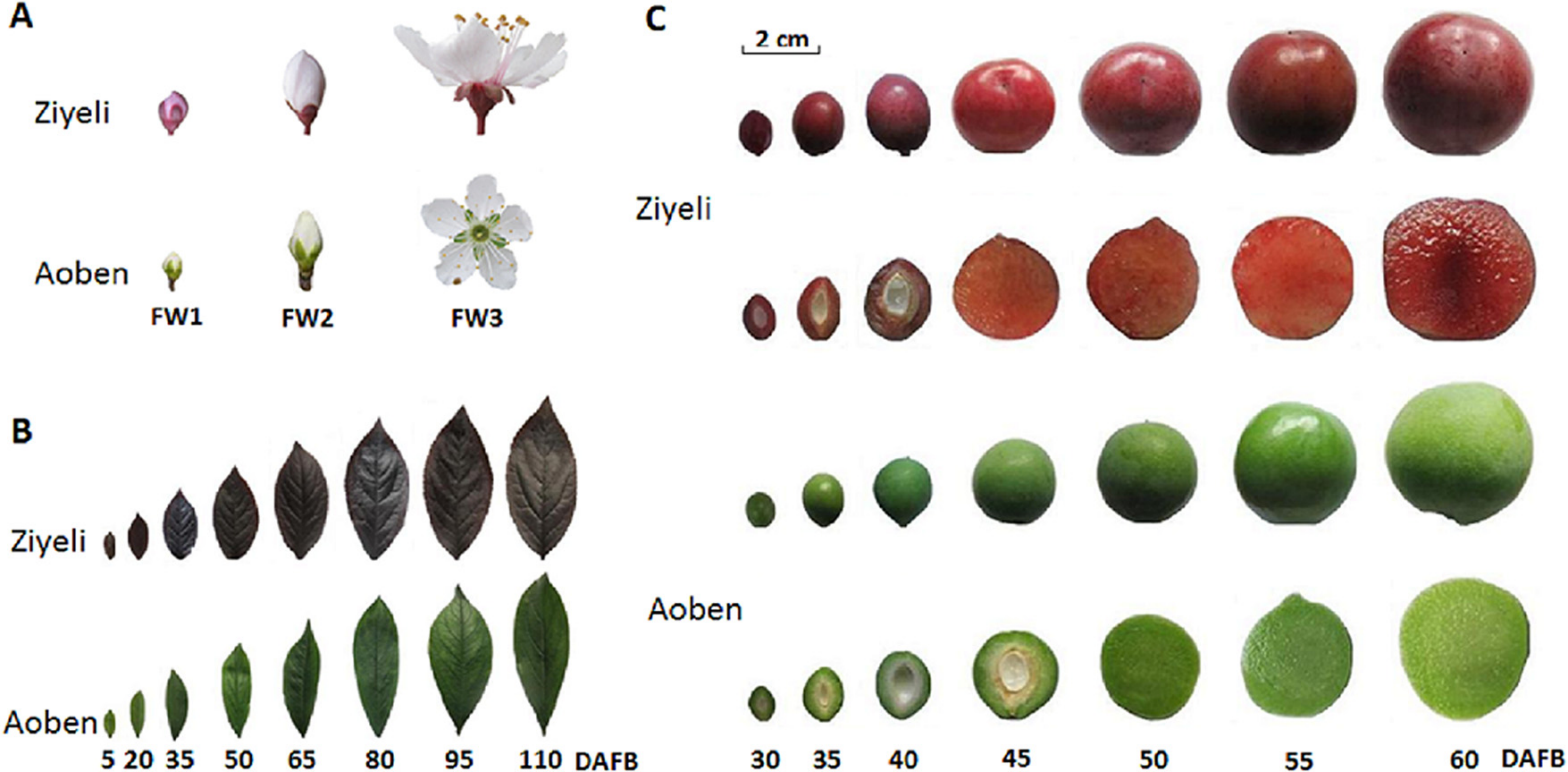
Pigmentation phenotype in flowers (A), leaves (B), and fruits (C) of two cherry plum cultivars Ziyeli and Aoben. FW1, flower buds at pink stage; FW2, flower buds at the bloom stage; FW3, flowers at full bloom. DAFB, days after full bloom.

Both ‘Ziyeli’ and ‘Aoben’ produce flowers with five petals and small drupes with 3–4 cm in diameter (Fig 1). In cultivar Ziyeli, red pigments are distributed throughout the fruit, from fruit set through maturity, and this phenotype is associated with dark red sepals and leaves. The flower petals are pink at the young bud stage, but the color of the petals fades to white after the bloom stage. In cultivar Aoben, however, green pigments instead of red pigments are distributed throughout the fruit, from fruit set through maturity, and this phenotype is associated with green leaves, white petals, and green sepals.

**Fig 2:**
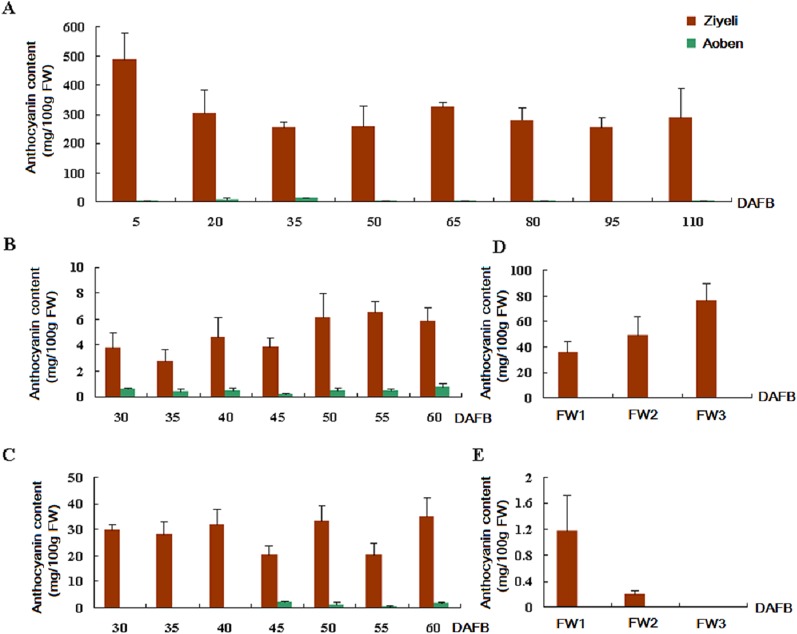
Anthocyanin content in leaf (A), fruit flesh (B) and skin (C), sepal (D), and petal (E) tissues of two cherry plum cultivars. FW1, flower buds at pink stage; FW2, flower buds at the bloom stage; FW3, flowers at full bloom.

Anthocyanin content in different tissues was measured for the two cherry plum cultivars according to our previously reported method [21]. In cv. Ziyeli, almost all the tested tissues, including leaf, fruit flesh and skin, and sepal, accumulated abundant anthocyanins (Fig 2). The anthocyanin accumulation in petal showed a decreasing trend, with undetectable at the full-bloom stage. In contrast, cv. Aoben accumulated extremely low levels of anthocyanins in all the tested tissues, and with no accumulation of anthocyanins in petal and sepal. In short, there is a significant difference in pigmentation phenotype between cv. Ziyeli and Aoben.

### Identification of anthocyanin biosynthetic genes in purple-leaf plum

To recover the coding sequences of anthocyanin biosynthetic genes, a mixture of leave samples of cv. Ziyeli, which were collected from every fifteen days from 5 to 110 days after full bloom, was chosen for RNA sequencing, and a total of 50.66 million pair-end clean reads in length of 100 bp were generated. Since the genomes of *Prunus* species are highly collinear [41, 42], these clean reads were mapped onto the peach reference genome [43]. The mapping result of transcriptome sequences revealed full-length coding sequences of anthocyanin structural genes, including *PcCHI*, *PcF3H*, *PcF3’H*, *PcDFR*, *PcLDOX*, *PcUFGT*, *PcCHS1*, and *PcCHS2*. However, only partial coding sequences were recovered for anthocyanin-activating *MYB* genes due to their low level of expression. There are six anthocyanin-activating *MYB* genes, *PpMYB10*.*1*-*PpMYB10*.*6*, in the peach genome [38]. Thus, six pairs of primers (S2 Table) flanking the whole coding regions of these six *PpMYB10* genes, respectively, were designed to amplify genomic DNA of cv. Ziyeli, and six anthocyanin-activating *MYB* genes designated *PcMYB10*.*1* through *PcMYB10*.*6* were successfully identified. The coding sequences of all these genes involved in anthocyanin biosynthesis in cv. Ziyeli were deposited in NCBI and the accession numbers are listed in S1 Table.

**Fig 3:**
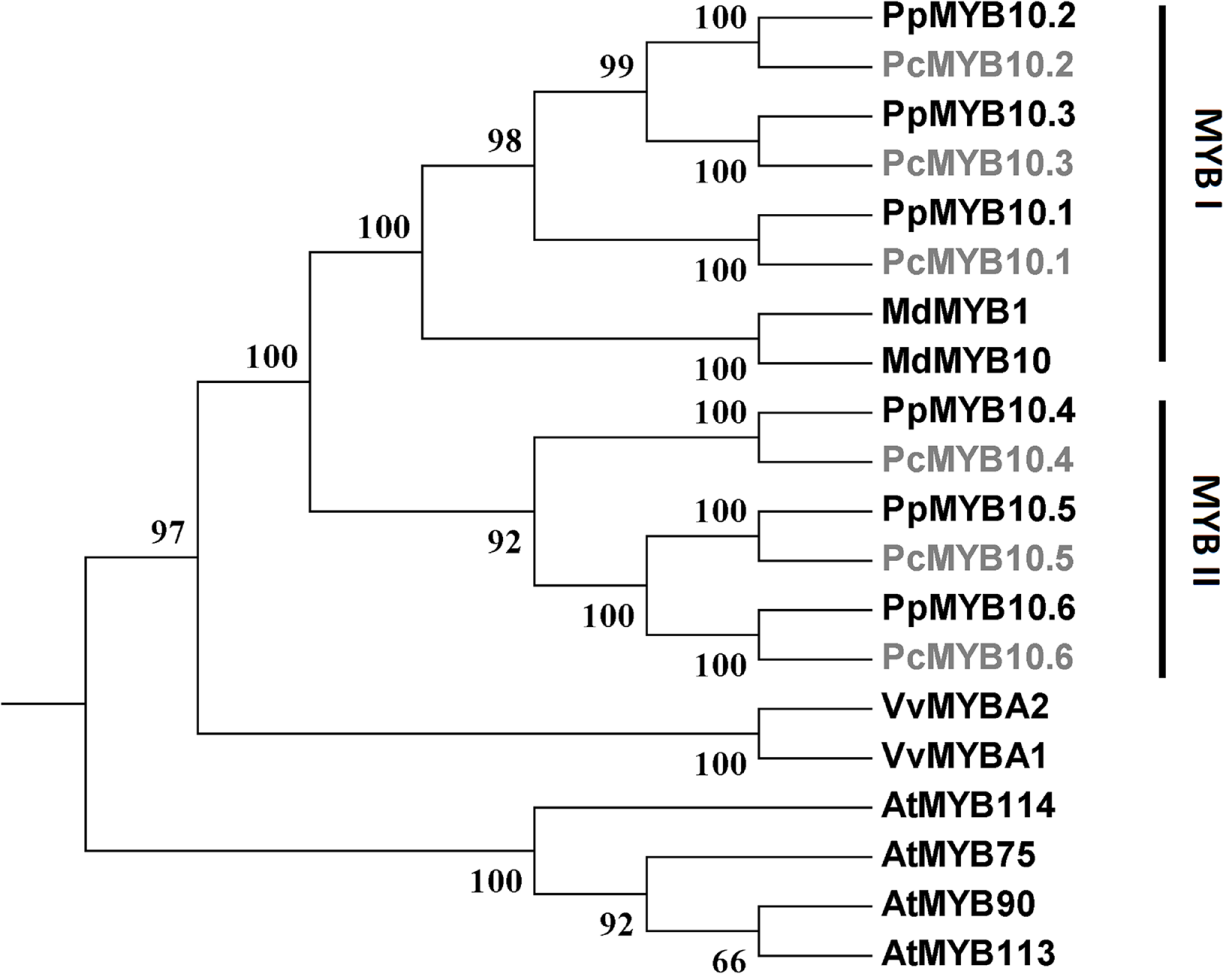
Phylogenetic tree derived from nucleotide acid sequence of anthocyanin-activating *MYB* genes in plants. Anthocyanin-activating *MYB* genes isolated in this study are highlighted in grey color. The GeneBank Accession numbers of these *MYB* genes are as follows: *Arabidopsis thaliana AtMYB75* (AF062908), *AtMYB90* (AF062915), *AtMYB113* (NM_105308), and *AtMYB114* (AY008379); *Malus × domestica MdMYB1* (AB744001) and *MdMYB10* (EU518249); *Prunus persica PpMYB10*.*1* (ppa026640m), *PpMYB10*.*2* (ppa016711m), *PpMYB10*.*3* (ppa020385m), *PpMYB10*.*4* (ppa018744m), *PpMYB10*.*5* (ppa024617m), and *PpMYB10*.*6* (ppa022808m); *Vitis vinifera VvMYBA1* (FJ687552) and *VvMYBA2* (DQ886419).

Phylogenetic analysis showed that the *PcMYB10* genes are closely related to previously reported anthocyanin-activating *MYB* genes in Roseceae, such as *MdMYB10* and *PpMYB10* (Fig 3). This result suggests that all the six *PcMYB10* genes are potential regulators that control anthocyanin pigmentation in purple-leaf plum. Thus, expression profiles of all the six *PcMYB10* genes and anthocyanin structural genes were subsequently investigated in different organs of two cultivars Ziyeli and Aoben. It is worth noting that *PcCHS1* and *PcCHS2* show a high level (93.9%) of identity in coding DNA sequence, thus, a common pair of primers were designed to investigate their collective expression.

### Expression profiling of anthocyanin structural genes and anthocyanin-activating *MYB* genes in leaves

**Fig 4:**
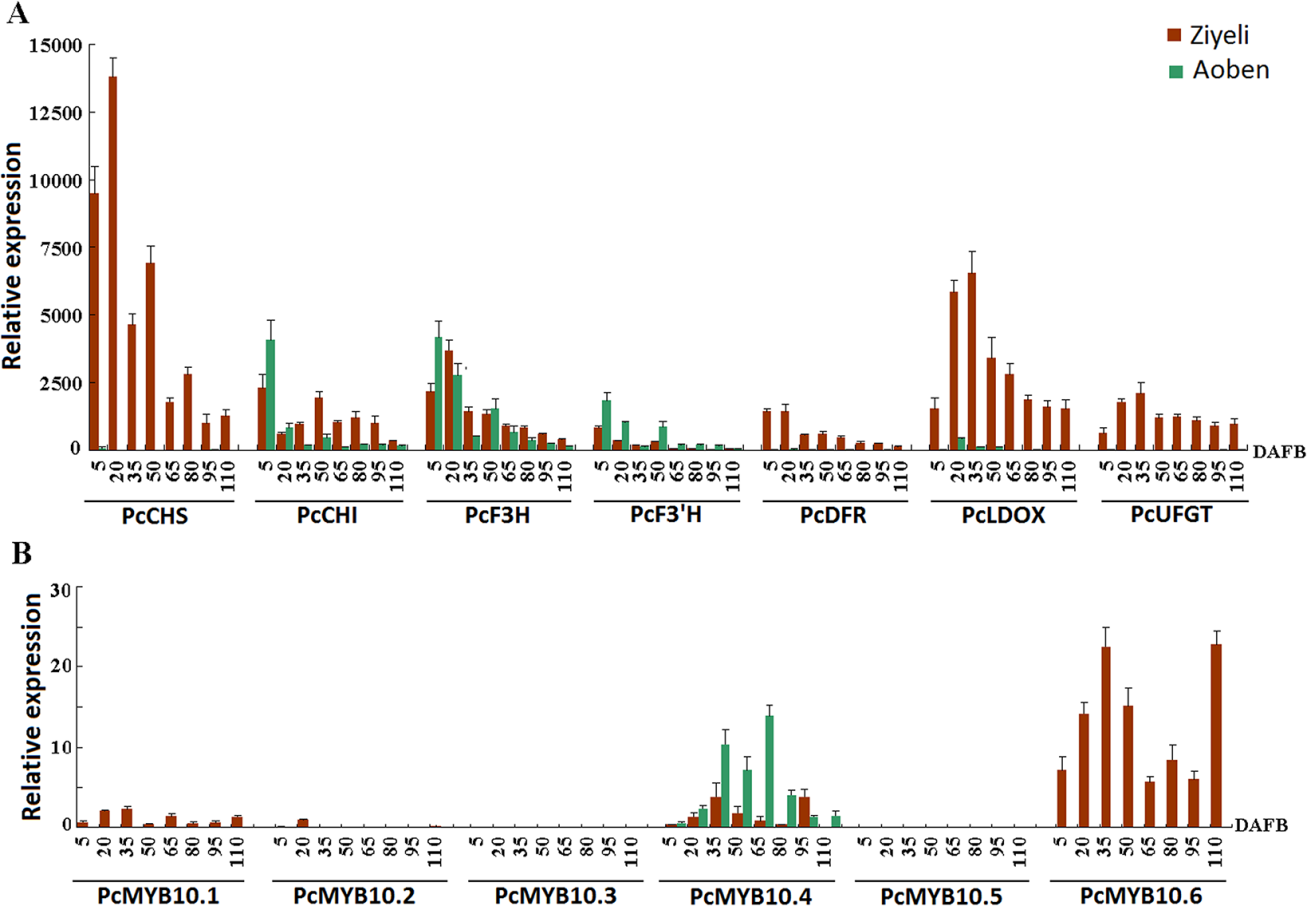
qRT-PCR analysis of the expression profiles of anthocyanin structural genes (A) and *PcMYB10* genes (B) in leaves of cv. Ziyeli and Aoben.

For anthocyanin structural genes, four genes, *PcCHS*, *PcDFR*, *PcLDOX*, and *PcUFGT*, were highly expressed in purple leaves of cv. Ziyeli during the whole course of leaf development, whereas their transcripts were almost undetectable in green leaves of cv. Aoben (Fig 4A). Three genes, *PcCHI*, *PcF3H*, and *PcF3’H*, were expressed in leaves of both cv. Ziyeli and Aoben. *PcCHI* and *PcF3H* showed higher level of expression in purple leaves than in green leaves, whilst the expression level of *PcF3’H* was significantly higher than that of green leaves. These results indicate that red pigmentation in cherry plum leaf is regulated at the transcriptional level.

Of the six *PcMYB10* genes, three (i.e. *PcMYB10*.*2*, *PcMYB10*.*3*, and *PcMYB10*.*5*) showed no or extremely low expression in both purple and green leaves (Fig 4B). *PcMYB10*.*4* gene was expressed in leaves, but showed higher level of expression in green leaves than in purple leaves. *PcMYB10*.*6* and *PcMYB10*.*1* were exclusively expressed in purple leaves, whilst the expression level of *PcMYB10*.*1* was significantly lower than that of *PcMYB10*.*6*. These results indicate that *PcMYB10*.*6* is likely responsible for purple coloration in leaves of cv. Ziyeli.

### Expression profiling of anthocyanin structural genes and anthocyanin-activating *MYB* genes in fruit flesh

**Fig 5:**
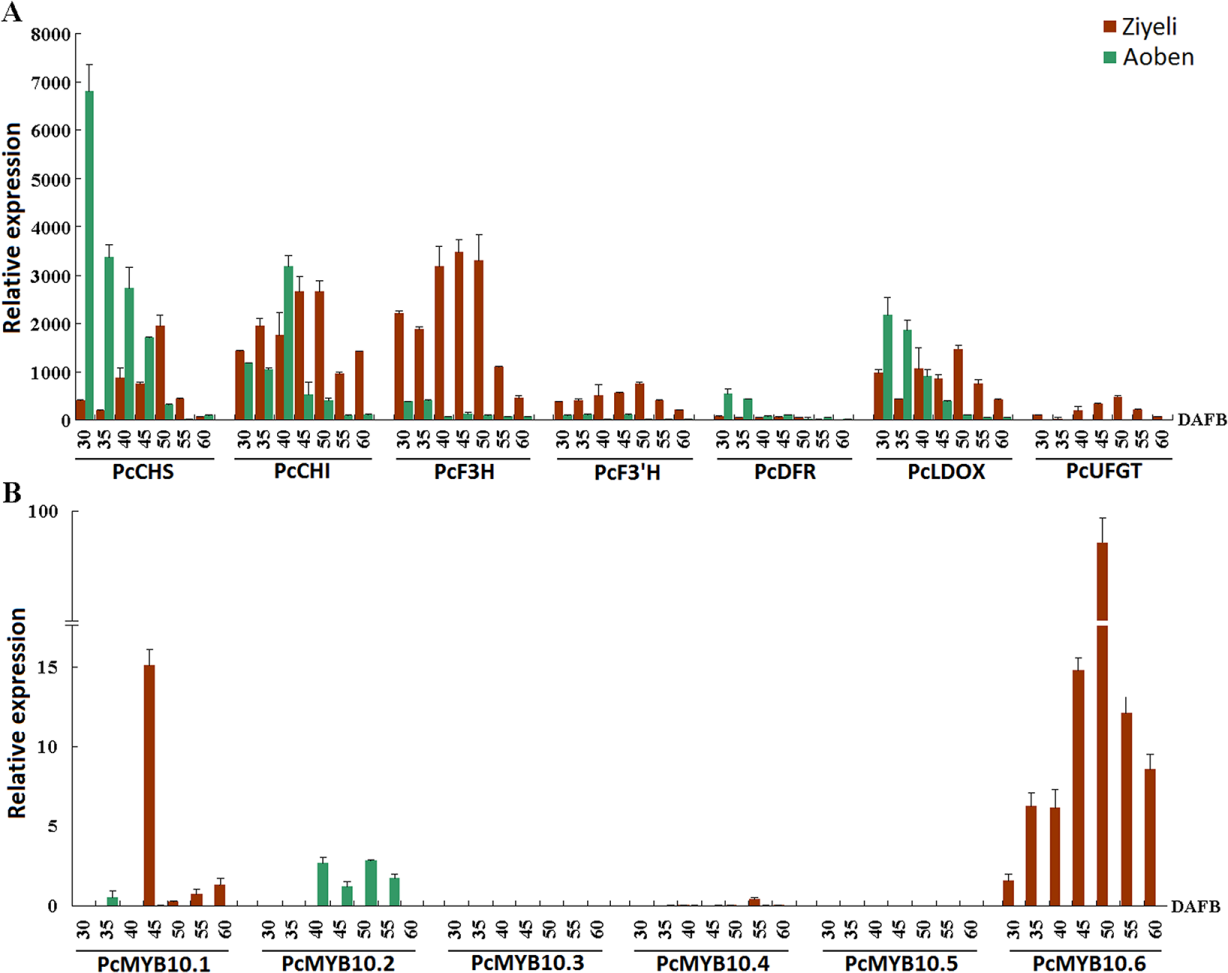
qRT-PCR analysis of the expression profiles of anthocyanin structural genes (A) and *PcMYB10* genes (B) in fruit flesh of cv. Ziyeli and Aoben.

Three anthocyanin structural genes, *PcUFGT*, *PcF3H*, and *PcF3’H*, were expressed in purple-coloured flesh, whilst their expression were extremely low or undetectable in green-coloured flesh (Fig 5A). *PcCHI* showed higher level of expression in purple-coloured flesh than in green-coloured flesh. *PcCHS* and *PcLDOX* were expressed in both purple- and green-coloured flesh, but their expression level was extremely low in green-coloured flesh during late stages of fruit development. *PcDFR* showed higher level of expression in green-coloured flesh than in purple-coloured flesh, but its expression level was very low when compared with other anthocyanin structural genes.

Among the six *PcMYB10* genes, three (i.e. *PcMYB10*.*3*, *PcMYB10*.*4*, and *PcMYB10*.*5*) were almost undetectable in both purple- and green-coloured flesh (Fig 5B). *PcMYB10*.*2* was expressed in green-coloured flesh during late stages of fruit development, whilst its transcripts were undetectable in purple-coloured flesh. In contrast, *PcMYB10*.*1* was expressed in purple-coloured flesh during late stages of fruit development, whist its transcripts were extremely low or undetectable in green-coloured flesh. *PcMYB10*.*6* was highly expression in purple-coloured flesh during the whole process of fruit development, whereas its transcripts were undetectable in green-coloured flesh. Thus, it seems that *PcMYB10*.*6* is the regulator controlling flesh purple coloration in cv. Ziyeli.

### Expression profiling of anthocyanin structural genes and anthocyanin-activating *MYB* genes in fruit skin

**Fig 6:**
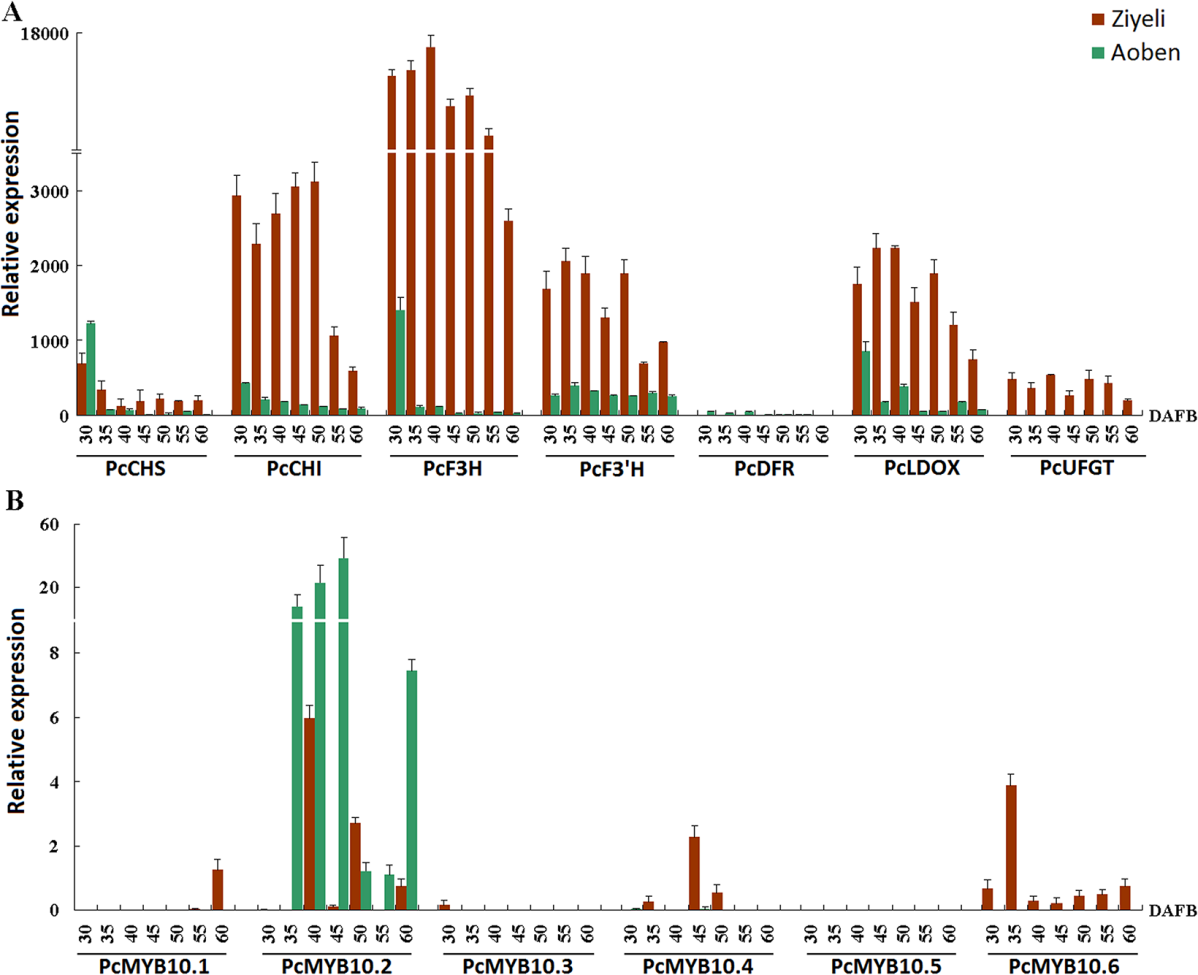
qRT-PCR analysis of the expression profiles of anthocyanin structural genes (A) and *PcMYB10* genes (B) in fruit skin of cv. Ziyeli and Aoben.

Five anthocyanin structural genes, *PcCHS*, *PcCHI*, *PcF3H*, *PcF3’H*, and *PcLDOX*, showed significantly higher level of expression in purple fruit skin than in green fruit skin (Fig 6A). *PcUFGT* was expressed in purple fruit skin, whereas its transcripts were undetectable in green fruit skin. The expression level of *PcDFR* was very low in both purple and green fruit skin when compared with other anthocyanin structural genes.

For the six *PcMYB10* genes, *PcMYB10*.*2* was highly expressed in both purple and green fruit skin, but its transcript level was significantly higher in green fruit skin than in purple fruit skin (Fig 6B). *PcMYB10*.*3* and *PcMYB10*.*5* were almost unexpressed in both purple and green fruit skin, and *PcMYB10*.*1* was expressed only in purple fruit skin at fruit ripening stage. *PcMYB10*.*6* was expressed in purple fruit skin during the whole process of fruit development. Thus, *PcMYB10*.*6* is likely responsible for purple coloration in fruit skin of cv. Ziyeli.

### Expression profiling of anthocyanin structural genes and anthocyanin-activating *MYB* genes in sepals

**Fig 7:**
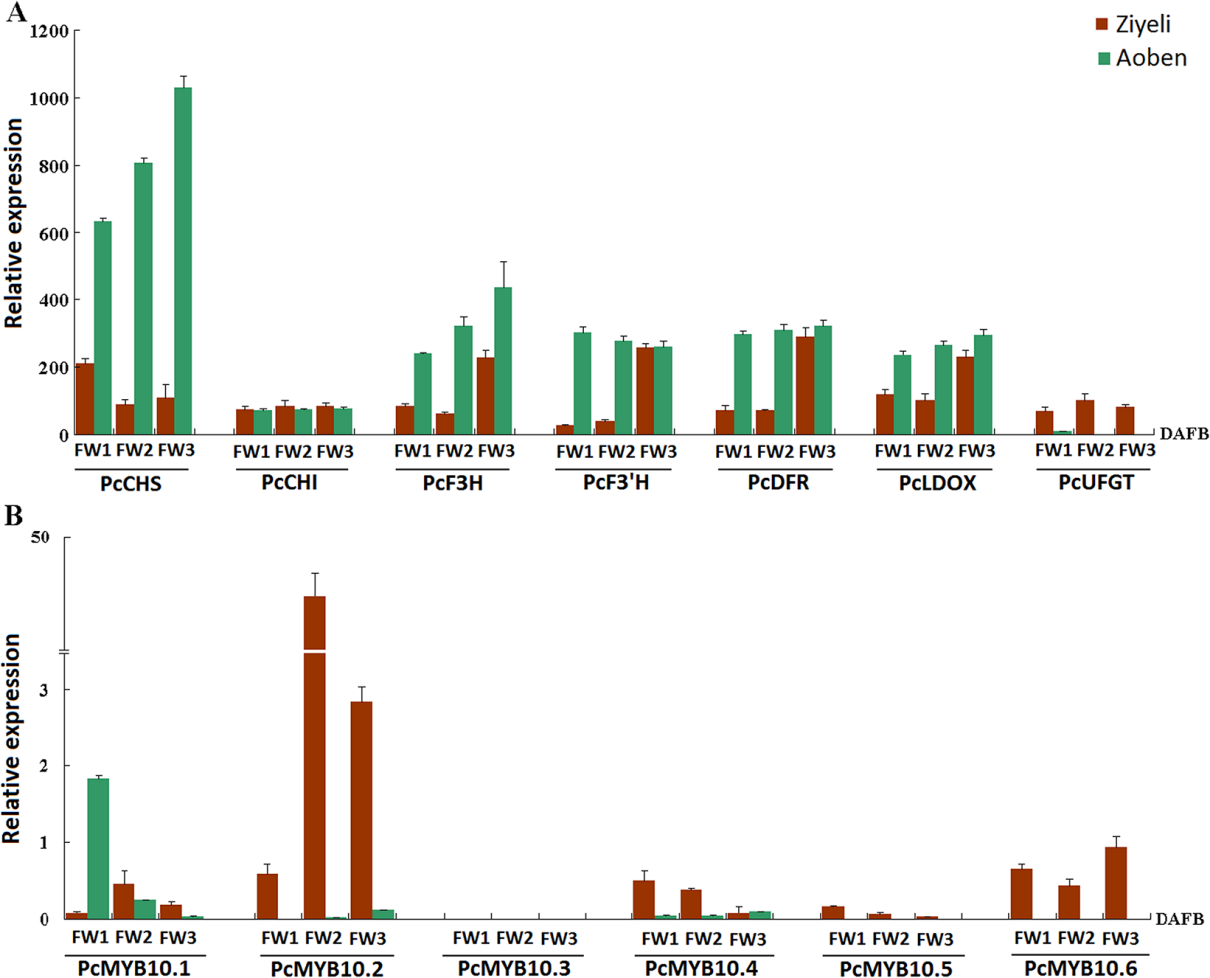
qRT-PCR analysis of the expression profiles of anthocyanin structural genes (A) and *PcMYB10* genes (B) in sepals of cv. Ziyeli and Aoben.

Most of anthocyanin structural genes, including *PcCHS*, *PcF3H*, *PcF3’H*, *PcDFR*, and *PcLDOX*, showed a higher level of expression in green sepals than in purple sepals, while the expression level of *PcCHI* was almost equal in purple and green sepals (Fig 7A). Only *PcUFGT* showed a significantly higher level of expression in red sepals than in the green sepals during the whole course of flower development.

Among the six *PcMYB10* genes, *PcMYB10*.*3* and *PcMYB10*.*5* were extremely low or undetectable in both green and purple sepals (Fig 7B). *PcMYB10*.*1* and *PcMYB10*.*4* showed an extremely low level of expression in red sepals at early and/or later stages of flower development. *PcMYB10*.*2* and *PcMYB10*.*6* were expressed in red sepals, and the expression level of *PcMYB10*.*2* in red sepals at late stages of flower development was significantly higher than that of *PcMYB10*.*6*. However, the transcript levels of *PcMYB10*.*2* and *PcMYB10*.*6* were undetectable or extremely low in green sepals. Thus, *PcMYB10*.*2* and *PcMYB10*.*6* genes may be related to purple coloration in red sepals of cv. Ziyeli.

### Expression profiling of anthocyanin structural genes and anthocyanin-activating *MYB* genes in petals

**Fig 8:**
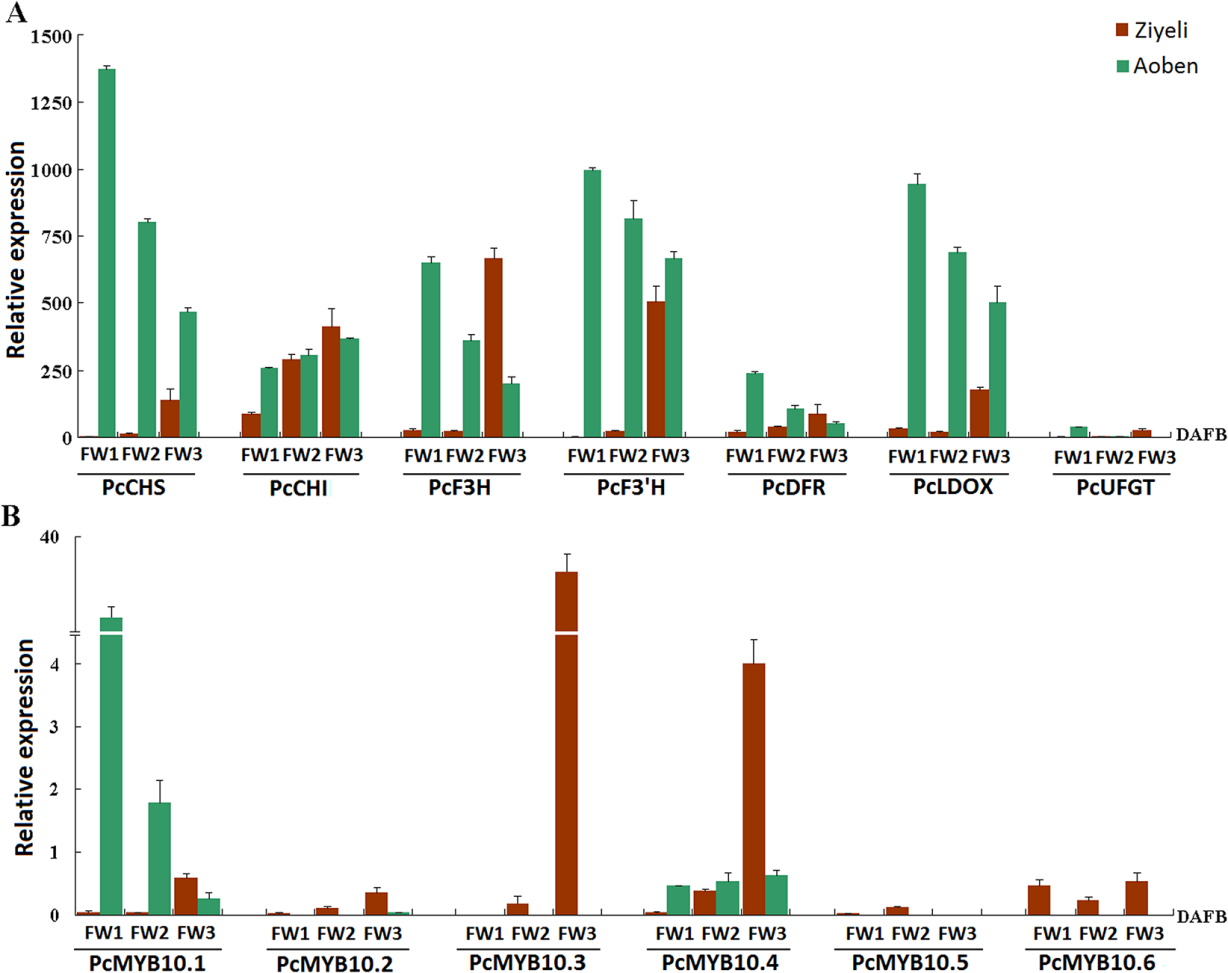
qRT-PCR analysis of the expression profiles of anthocyanin structural genes (A) and *PcMYB10* genes (B) in petals of cv. Ziyeli and Aoben.

During early stages of flower development, all the anthocyanin structural genes showed higher level of expression in petals of cv. Ziyeli than in petals of cv. Aoben. Interestingly, the expression level of *PcUFGT* was extremely low in both pink and white petals when compared with other anthocyanin structural genes (Fig 8A).

For the six *PcMYB10* genes, *PcMYB10*.*2* and *PcMYB10*.*5* showed an extremely low level of expression in petals of cv. Ziyeli and Aoben (Fig 8B). *PcMYB10*.*1* was highly expressed in petals of cv. Aoben, whilst its transcript level was extremely low in pink petals of cv. Ziyeli. *PcMYB10*.*3* and *PcMYB10*.*4* were extremely low in petals of cv. Ziyeli at flower bud stage, whereas their transcripts were abundant in petals at full-bloom stage. *PcMYB10*.*6* was exclusively expressed in petals of cv. Ziyeli during the whole process of flower development. Thus, all the six *PcMYB10* genes are unlikely responsible for flower red coloration in cv. Ziyeli.

## Discussion

In Rosaceae, red coloration is mainly due to the activation of MYB-type anthocyanin regulators [37]. Here, six *PcMYB10*.*6* genes were identified in cherry plum, and the association of their expression with red coloration was investigated. In the wild-type cultivar Aoben, *PcMYB10*.*1*, *PcMYB10*.*2*, and *PcMYB10*.*4* are expressed in flowers, fruits, or leaves, whilst the transcripts of *PcMYB10*.*3*, *PcMYB10*.*4*, and *PcMYB10*.*6* are undetectable in all the tested organs. In purple-leaf cv. Ziyeli, five *PcMYB10* genes (*PcMYB10*.*1* through *PcMYB10*.*5*) show distinct spatial and temporal expression patterns, whilst *PcMYB10*.*6* is highly expressed in leaves, flowers, and fruits throughout all tested developmental stages. This indicates that the expression of *PcMYB10*.*6* is constitutively activated in cv. Ziyeli. *PcMYB10*.*6* gene belongs to the MYBII family which regulates anthocyanin pigmentation in vegetative organs (Fig 3), but its constitutive activation results in red pigmentation in both foliage and fruit (skin and cortex). A similar case is also observed for the apple *MdMYB10* gene. *MdMYB10* is a member of the MYBI family regulating anthocyanin pigmentation in fruits, but its constitutive expression causes red pigmentation in both fruit and foliage. In apple, the constitutive expression of *MdMYB10* is due to duplication of a 23-bp sequence motif in the promoter that is a target of the MdMYB10 protein itself [44]. Since apple and plum belong to the same family of Rosaceae, it is worth of further study to address whether the constitutive activation of *PcMYB10*.*6* in purple-leaf plum is due to the same mechanism underlying the constitutive expression of *MdMYB10* in the red-fleshed apple ‘Red Field’.

It is worth noting that *PcMYB10*.*2* is highly expressed in fruit skin of cherry plum cv. Aoben, and its expression level is significantly higher than the expression level of *PcMYB10*.*6* in fruit skin of purple-leaf plum. However, high-level expression of *PcMYB10*.*2* cannot induce red pigmentation in fruit skin. Likewise, *PcMYB10*.*2* is expressed in fruit flesh of cv. Aoben, but its expression cannot induce red pigmentation in fruit flesh either. In sepals of cv. Ziyeli, *PcMYB10*.*2* is highly expressed and its expression level reaches a peak at the bloom stage. In contrast, most anthocyanin structural genes such as *PcF3H*, *PcF3’H*, *PcDFR*, and *PcLDOX* show relatively low levels of expression in sepals at early stages of flower development, and their expression levels increase significantly at full-bloom stage. This inconsistency between the *PcMYB10*.*2* expression and the induction of anthocyanin biosynthetic genes indicates *PcMYB10*.*2* is unlikely involved in the regulation of red pigmentation in the sepal of cherry plum. In addition, three tandem-duplicated *MYB* genes, *PpMYB10*.*1* to *PpMYB10*.*3*, are located on chromosome 3 of peach [38]. All these three *PpMYB10* genes belong to the MYBII family, but *PpMYB10*.*2* is not involved in anthocyanin pigmentation in either the flesh or skin of fruits [18]. Moreover, our recent study shows the *PpMYB10*.*2* expression alone is unable to induce anthocyanin pigmentation in peach leaf [38]. Taken together, all the results above suggest that functional divergence may occur after the duplication of *MYB10* genes in *Prunus*, and the two orthologs *PcMYB10*.*2* and *PpMYB10*.*2* play little role in regulation of anthocyanin biosynthesis.

In peach, anthocyanin-activating *MYB* gene *PpMYB10*.*4* is involved in the regulation of anthocyanin pigmentation in leaf [38]. The cherry plum *PcMYB10*.*4* is identical in amino acid sequence to *PpMYB10*.*4* (S2 Fig). In cherry plum cv. Aoben, *PcMYB10*.*4* is expressed in foliage, but its expression alone cannot induce red pigmentation due to the lack of expression of several anthocyanin structural genes such as *PcCHS* and *PcUFGT*. It has been reported that MYB repressors may compete with MYB activators for binding sites of bHLH and/or anthocyanin structural genes [45–47]. Thus, further studies are needed to clarify whether anthocyanin pigmentation in foliage of cherry plum is coordinatively regulated by both positive and negative regulators of anthocyanin biosynthesis, and whether the negative regulators repress both anthocyanin structural and regulatory genes.

Anthocyanin-related MYB genes are well known to be involved in the regulation of flower coloration in plants such as *Antirrhinum majus* [48], *Gerbera hybrida* [49], *Nicotiana tabacum* [50], *Oncidium* [51], *Petunia hybrida* [52, 53] and *Pisum sativum* [54]. More recently, an R2R3 MYB gene termed PEACE has been shown to be able to induce the expression of anthocyanin biosynthetic genes, resulting in red pigmentation in the petal of flowering peach [55]. In this study, all the anthocyanin structural genes except *PcUFGT* show higher levels of expression in both petal and sepal of cv. Ziyeli than in those of cv. Aoben. This indicates that induction of *PcUFGT* gene, which catalyzes the last step in anthocyanin biosynthesis, is crucial for red pigmentation in flower of cherry plum. In sepals of cv. Ziyeli, the expression of the *PcMYB10*.*6* gene is consistent with the induction of the *PcUFGT* gene. Thus, *PcMYB10*.*6* is likely involved in the regulation of red pigmentation in the sepal of cherry plum. In petals of cv. Ziyeli, *PcMYB10*.*6* gene is constitutively active, whilst the transcript level of *PcUFGT* gene is almost undetectable during the whole process of flower development. It is unclear why *PcMYB10*.*6* fails to induce the expression of *PcUFGT* gene in petal although it is able to induce the expression of *PcUFGT* gene in leaf, fruit (skin and flesh), and sepal. It is well known that *MYB* regulators require a bHLH3 partner to activate transcription of anthocyanin structural genes in plants of the Rosaceae family, such as peach [56] and apple [5, 37, 44]. Thus, we isolated *PcbHLH3* in plum and its expression profile was also investigated. However, the *PcbHLH3* gene was highly expressed in all the tested tissues throughout development in both ‘Ziyeli’ and ‘Aoben’. Thus, the failure of transcriptional activation of *PcUFGT* in petal cannot be attributed to the *PcbHLH3* gene.

Anthocyanidin reductase is well known to compete with the UFGT enzyme to convert anthocyanidin to epicatechin, resulting in redirection of anthocyanin pathway into proanthocyanidin pathway [31, 33, 34, 57]. Therefore, we further investigated the expression profile of *PcANR* in petals (S3 Fig). The expression level of *PcANR* was extremely low in petals of cv. Ziyeli at pink stage and increased significantly at full-bloom stage, while *PcANR* was constitutively highly expressed in petals of cv. Aoben during the whole process of flower development. This result suggests that the colour varies from pink to white in the petal of cv. Ziyeli probably results from the increased expression of *PcANR* during later stages of flower development (e.g., bloom and full bloom). In the petal of cv. Aoben, constitutive over-expression of *PcANR* may cause anthocyanidin flux to proanthocyanidin pathway, which in turn blocks the biosynthesis of anthocyanin. In addition, it is unclear whether the high-level expression of both *PcANR* and all anthocyanin structural genes except *PcUFGT* in the petal and sepal of cv. Aoben is induced by regulatory flavonoid genes.

Taken together, constitutive activation of *PcMYB10*.*6* is related to red pigmentation in floral and vegetative tissues of cherry plum, including leaf, fruit (flesh and skin), and sepal. However, the mechanism underlying constitutive activation of *PcMYB10*.*6* remains unclear. In the petal of cherry plum, the inhibition of red pigmentation could be attributed to the high-level expression of *PcANR* that directs anthocyanidin flux to proanthocyanidin biosynthesis.

## Supporting Information

S1 Table Primers for qRT-PCR analyses in cherry plum. (DOC)

S2 Table The specific primer sets for *PcMYB10*.*1* through *PcMYB10*.*6*. (DOC)

S1 Fig Expression profiling of the *PcGAPDH* gene in leaf, fruit flesh and skin, sepal, and petal tissues using semi-quantitive RT-PCR. (DOC)

S2 Fig Amino acid sequence alignment of anthocyanin-activating MYB genes in plum, peach and apple. The R2 and R3 repeats of the MYB DNA binding domain are boxed. Conserved amino acid sequences are indicated by a black ground and similar amino acids by a light gray background.
(DOC)

S3 Fig qRT-PCR analysis of the expression levels of *PcANR* gene in petals of two cherry plum cv. Ziyetao and Aoben.
(DOC)

S4 Fig Expression profiling of *PcbHLH3* genes in different tissues of cv. Ziyeli and Aoben using qRT-PCR.
(DOC)
