# Supplementary material for: Constitutive Activation of an Anthocyanin Regulatory Gene PcMYB10.6 Is Related to Red Coloration in Purple-Foliage Plum
Source: PLoS One. 2015 Aug 6;10(8):e0135159. doi: 10.1371/journal.pone.0135159 (PMC4527586; doi:10.1371/journal.pone.0135159)
Supplement: S1 Table — (DOC) [file pone.0135159.s001.doc]

S1 Table. Primers for qRT-PCR analyses in cherry plum

| Name | Accession no. | Forward (5’→3’) | Reverse (5’→3’) |
| --- | --- | --- | --- |
| *PcCHS1/PcCHS2* | KP772275/KP772276 | CATCCGTCAAGCGCCTCAT | GGTCCGCACCCACAATAATAGC |
| *PcCHI* | KP772274 | TTACACTGATGCAGAAGCCAAGG | AAAGCAGTTTATTTTCGATCACCAC |
| *PcF3H* | KP772278 | GGATGGTGGGAAGACGTGGAT | CTGGGTTCTGGAATGTGGCTATG |
| *PcF3’H* | KP772279 | GCTCAAAGAGGATGCTGACGGT | GGTATGTCCAGTTCAGTTACAAGCC |
| *PcDFR* | KP772277 | TCTTCCCATGATGCTACAATTTACG | GAGACTTTGACATCGACGACGGT |
| *PcLDOX* | KP772280 | ATACCCTGAGGACAAGCGTGAC | CCAATCCCAACCCAAGTGACA |
| *PcUFGT* | KP772287 | TTGGCTCCCTCTTTGGCTCT | GTTGACATTCTTGGTGATGAGTTCG |
| *PcANR* | KP772273 | AAAGTTCCTCAACGAAAGATACCCT | TCGTACTTAAAGTCGAATCCCTCCT |
| *PcMYb10.1* | KP772281 | TCCAGGAAGGACAGCGAATG | TGTTGATGGTGATGTTTGTGACG |
| *PcMYB10.2* | KP772282 | CCCGATTGCGGACGGATTAT | AACCCAAGACCAGAACCTGTAGC |
| *PcMYB10.3* | KP772283 | CAATAAAGCCCATCGTCACAAG | TCATCAAACCCAGGACCAGAAC |
| *PcMYB10.4* | KP772284 | AACTGCCAATACTACCCTCATCG | GTCCACGGAGCCAAATCTTCA |
| *PcMYB10.5* | KP772285 | TGTTACCTTCTGTCTTCCCTTCATA | ACCGTACTGTTGAATGTATTGCCTG |
| *PcMYb10.6* | KP772286 | TCCCTTTGTATGGAGAGCAGAGATG | CCGTACTGTTGAATGTATTGCCTG |
| *PcGAPDH* | KP765685 | CAATGCCATTCAAGCTAAGG | GAAATTCGATTTGCATGAGC |
| *PcbHLH3* | KT254006 | ACCACAGTTGTCCAGTCCTCATTG | TACCCTCTTCACTTCCGTAATGC |
