# Supplementary material for: Constitutive Activation of an Anthocyanin Regulatory Gene PcMYB10.6 Is Related to Red Coloration in Purple-Foliage Plum
Source: PLoS One. 2015 Aug 6;10(8):e0135159. doi: 10.1371/journal.pone.0135159 (PMC4527586; doi:10.1371/journal.pone.0135159)
Supplement: S2 Table — (DOC) [file pone.0135159.s002.doc]

S2 Table. The specific primer sets for *PcMYB10.1* through *PcMYB10.6*

| Genes | Forward (5’→3’) | Reverse (5’→3’) |
| --- | --- | --- |
| *PcMYB10.1* | ACGCTGGCCAACAAGAAGATG | CCGCTCTAAAAAGCAACCTCATC |
| *PcMYB10.2* | CATAACGCTGGCCAAGACACC | CAGTTGCTGATAATTGCTACTAGGAAG |
| *PcMYB10.3* | AAGCATACAACGCTGGCTAAAAG | AGTAACCCGACTTCTTATAAACCCAG |
| *PcMYB10.4* | CGGAGGGTTTGAGGATGTTGGC | CGTTGAAATAAAATTCATAGTTGGTCCC |
| *PcMYB10.5* | TTAGACAGGTTGTGTCTGGAGGCT | TGTTTAAAAAGGGATGCATAAGAGTTC |
| *PcMYB10.6* | GAGAGGAGAGGTGCACGGAAG | TGGGAAGACATAGACCCTCTGAACC |
