# Supplementary material for: Constitutive Activation of an Anthocyanin Regulatory Gene PcMYB10.6 Is Related to Red Coloration in Purple-Foliage Plum
Source: PLoS One. 2015 Aug 6;10(8):e0135159. doi: 10.1371/journal.pone.0135159 (PMC4527586; doi:10.1371/journal.pone.0135159)
Supplement: S1 Fig — (DOC) [file pone.0135159.s003.doc]

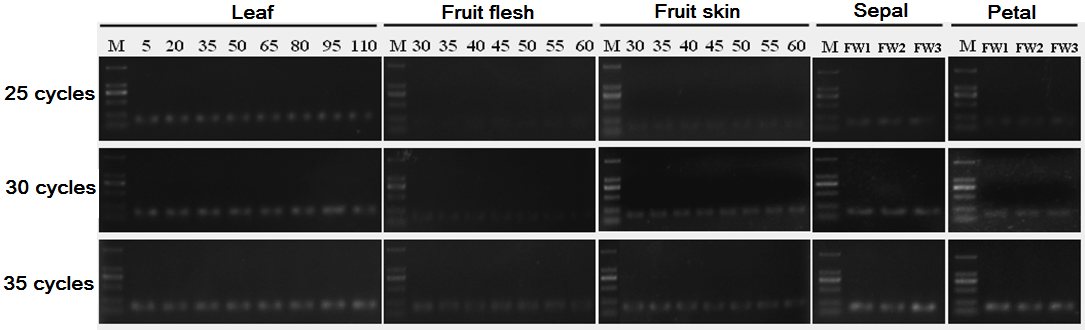


S1 Fig. Expression profiling of the *PcGAPDH* gene in leaf, fruit flesh and skin, sepal, and petal tissues using semi-quantitive RT-PCR.
