# Supplementary material for: Constitutive Activation of an Anthocyanin Regulatory Gene PcMYB10.6 Is Related to Red Coloration in Purple-Foliage Plum
Source: PLoS One. 2015 Aug 6;10(8):e0135159. doi: 10.1371/journal.pone.0135159 (PMC4527586; doi:10.1371/journal.pone.0135159)
Supplement: S2 Fig — The R2 and R3 repeats of the MYB DNA binding domain are boxed. Conserved amino acid sequences are indicated by a black ground and similar amino acids by a light gray background. (DOC) [file pone.0135159.s004.doc]

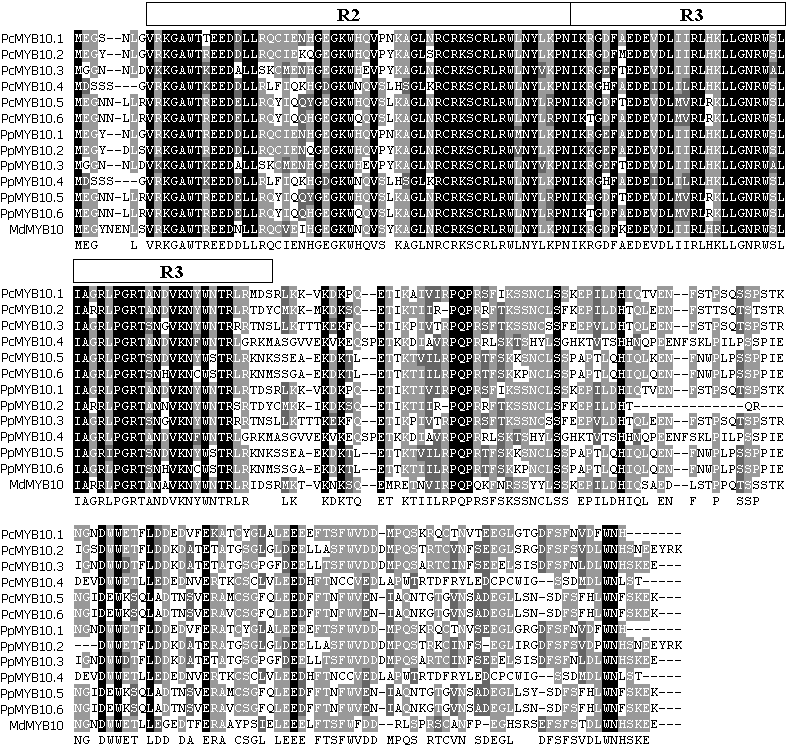


S2 Fig. Amino acid sequence alignment of anthocyanin-activating MYB genes in plum, peach and apple. The R2 and R3 repeats of the MYB DNA binding domain are boxed. Conserved amino acid sequences are indicated by a black ground and similar amino acids by a light gray background.
