# Supplementary material for: Constitutive Activation of an Anthocyanin Regulatory Gene PcMYB10.6 Is Related to Red Coloration in Purple-Foliage Plum
Source: PLoS One. 2015 Aug 6;10(8):e0135159. doi: 10.1371/journal.pone.0135159 (PMC4527586; doi:10.1371/journal.pone.0135159)
Supplement: S3 Fig — Ziyetao and Aoben. (DOC) [file pone.0135159.s005.doc]

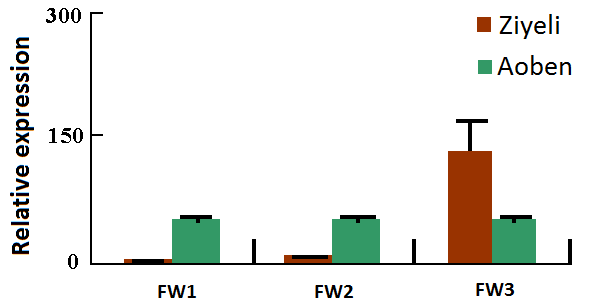


S3 Fig. qRT-PCR analysis of the expression levels of *PcANR* gene in petals of two cherry plum cv. Ziyetao and Aoben.
