# Supplementary material for: Constitutive Activation of an Anthocyanin Regulatory Gene PcMYB10.6 Is Related to Red Coloration in Purple-Foliage Plum
Source: PLoS One. 2015 Aug 6;10(8):e0135159. doi: 10.1371/journal.pone.0135159 (PMC4527586; doi:10.1371/journal.pone.0135159)
Supplement: S4 Fig — Ziyeli and Aoben using qRT-PCR. (DOC) [file pone.0135159.s006.doc]

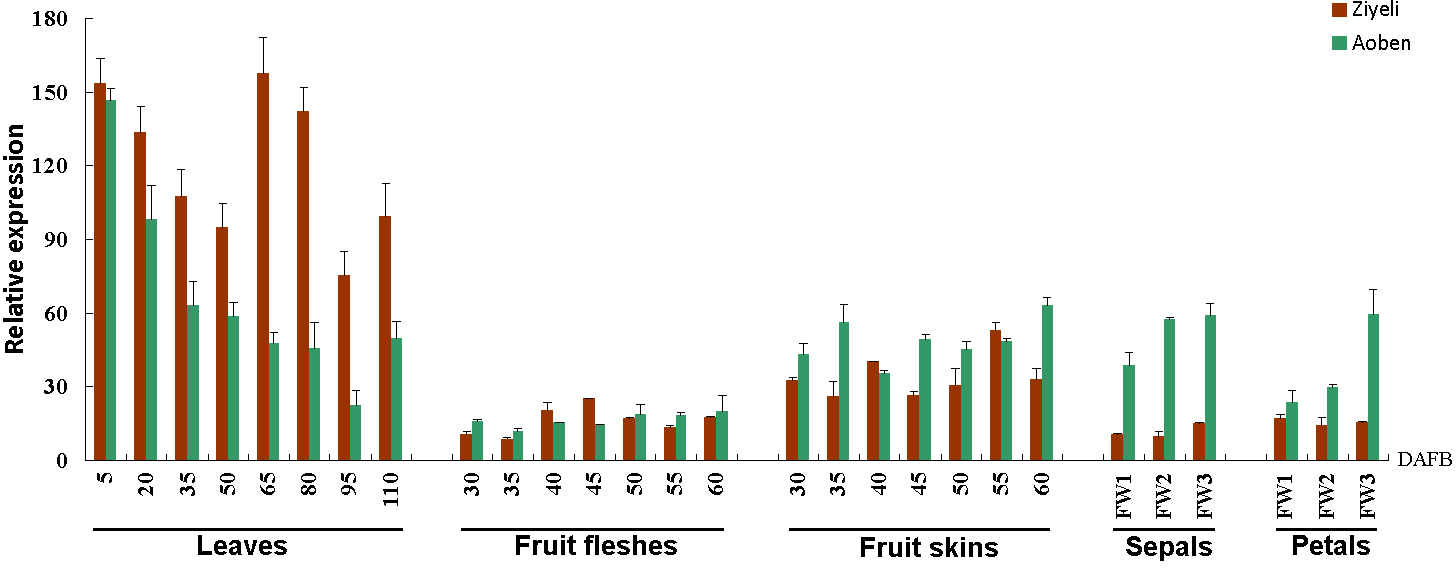


S4 Fig. Expression profiling of *PcbHLH3* genes in different tissues of cv. Ziyeli and Aoben using qRT-PCR.
